# Pilot turning behavior cognitive load analysis in simulated flight

**DOI:** 10.3389/fnins.2024.1450416

**Published:** 2024-09-23

**Authors:** Wen-gang Zhou, Pan-pan Yu, Liang-hai Wu, Yu-fei Cao, Yue Zhou, Jia-jun Yuan

**Affiliations:** Flight Technology College, Civil Aviation Flight University of China, Guanghan, China

**Keywords:** safe ergonomics, cognitive load, turning behavior, heart rate variability, simulated flight

## Abstract

**Background:**

To identify the cognitive load of different turning tasks in simulated flight, a flight experiment was designed based on real “preliminary screening” training modules for pilots.

**Methods:**

Heart Rate Variability (HRV) and flight data were collected during the experiments using a flight simulator and a heart rate sensor bracelet. The turning behaviors in flight were classified into climbing turns, descending turns, and level flight turns. A recognition model for the cognitive load associated with these turning behaviors was developed using machine learning and deep learning algorithms.

**Results:**

pnni_20, range_nni, rmssd, sdsd, nni_20, sd1, triangular_index indicators are negatively correlated with different turning load. The LSTM-Attention model excelled in recognizing turning tasks with varying cognitive load, achieving an F1 score of 0.9491.

**Conclusion:**

Specific HRV characteristics can be used to analyze cognitive load in different turn-ing tasks, and the LSTM-Attention model can provide references for future studies on the selection characteristics of pilot cognitive load, and offer guidance for pilot training, thus having significant implications for pilot training and flight safety.

## 1 Introduction

A pilot’s cognitive load refers to the cognitive resources allocated to attending, perceiving, making decisions, and acting essentially, the total workload and energy required to process information per unit of time. Cognitive Load Theory (CLT) explains how cognitive resources are allocated during information processing. CLT emphasizes that working memory has a limited capacity, and as task complexity and the amount of information increase, cognitive resource consumption also increases, leading to a cognitive load. With the increasing complexity of human-computer interaction systems in aircraft piloting, pilots face a growing cognitive load during operations (Jinsong, 2019). Studies indicate excessive cognitive load can cause pilots to miss critical situational information (Wang et al., 2023). An illustrative case is Air Asia Flight 8501’s crash, where pilots misjudged the aircraft’s attitude, position, and motion during a turning maneuver, resulting in catastrophic failure. Given pilots’ limited information processing capacity, simultaneously receiving data from multiple sources can lead to ‘information overload.’ This overload can exacerbate cognitive load, adversely affect performance, and pose significant flight safety risks. Current research on pilot cognitive load focuses predominantly on takeoff and landing, with turning maneuvers receiving minimal attention (Meng and LiMing, 2019). During turns, pilots must simultaneously manage the control stick and rudders and monitor safety parameters like attitude, altitude, and more. In these scenarios, the cognitive load intensifies, often leading to notable physiological responses, including significant Heart Rate (HR) changes (Jingzhou, 2018).

Traditionally, pilot cognitive load assessment has relied on subjective scales. For example, pilot workload can be quantified across various task levels during flight approach via the NASA-TLX subjective scales (Zhang et al., 2015). Additionally, the effectiveness of both the NASA-TLX scale and the Modified Cooper-Harper scale in gauging the mental load of pilots within a flight training context has been demonstrated (Mansikka et al., 2019). However, this approach has notable limitations. It requires pilots to perform assessments at specific intervals, fails to provide continuous monitoring data, and individual perceptions and varying environmental conditions largely influence results. These factors complicate accurate reflection of pilots’ actual working conditions. Moreover, the dynamic and complex flight environment demands pilots make rapid decisions, underscoring the need for realtime monitoring of pilots’ physiological and psychological states. In this paper, heart rate monitoring via a wearable device allows for noninvasive, continuous physiological data collection during flight without interfering with the pilot’s normal operations.

Pilot cognitive load can be effectively measured using HRV, an objective physiological indicator reflecting the autonomic nervous system balance between sympathetic and parasympathetic activities. Moreover, serves as an objective physiological indicator widely used to evaluate an individual’s stress levels and cognitive load. This measure reflects the balance between sympathetic and parasympathetic activity within the autonomic nervous system (ANS). HRV embodies the interaction between the nervous system and cardiac activity, influenced by dynamic changes in the ANS and physical and environmental factors such as temperature, respiration, hormones, and blood pressure. During complex or stressful flight missions, pilots must process large amounts of information, make rapid decisions, and maintain intense focus. This elevated cognitive load activates the sympathetic nervous system, leading to a decrease in HRV. This decline indicates the pilot’s current physiological stress and the depletion of their cognitive resources. As mission complexity increases, the drop in HRV becomes more pronounced, suggesting that the pilot is under a higher cognitive load. Conversely, in more straightforward or familiar mission scenarios, the pilot’s cognitive load is lower, leading to increased parasympathetic activity and a corresponding rise in HRV. This indicates that the pilot has more cognitive resources for other tasks, reducing the overall stress. HRV effectively captures pilots’ sympathetic stress responses across various flight conditions, whether in actual or simulated environments. By monitoring HRV changes, a comprehensive understanding of the pilot’s stress and cognitive load can be achieved, making HRV a vital reference index for evaluating pilot performance and well-being. HRV captures sympathetic stress responses of pilots across various flight conditions in actual and simulated environments. For instance, HRV was analyzed in 34 pilots to gauge workload across different flight phases—takeoff, steady turn, landing—, noting distinct patterns. However, changes in the low-frequency to high-frequency ratio were insignificant (Xiaohua et al., 2023; Alaimo et al., 2022). HRV combined with machine learning algorithms (SVM, KNN, LDA) was used to assess cognitive load in fighter jet pilots across flight stages (Mohanavelu et al., 2022). HR and HRV were monitored in fighter pilots to investigate cognitive load and performance differences under varying task conditions (Mansikka et al., 2016). Physiological signals were also utilized to identify cognitive demands on pilots, observing that increased training task difficulty escalated cognitive load and corresponding physiological changes (Mouratille et al., 2018). An index using ECG data and principal component analysis was developed to identify high cognitive load during simulated flights (Jinghua et al., 2023). Research on pilot cognitive load has traditionally overlooked phases like leveling and turning, critical for recognizing pilot load levels during flight maneuvers. To address this gap, a simulated flight environment was established that mimicked tasks such as turning at various angles. This setup enabled the collection of real-time data from flight trainees, including flight data and HRV, using flight simulators equipped with HR-monitoring bracelets. Statistical methods such as the Kolmogorov-Smirnov normality test, Analysis of Variance (ANOVA), and the Kruskal-Wallis (K-W) test were employed to pinpoint significant characteristics in the data. The cognitive load of pilots during various turning tasks was analyzed and accurately identified using a combination of machine learning and deep-learning algorithms. This innovative approach not only aids in better understanding pilot cognitive load but also contributes significantly to pilot health management, load assessment, and overall flight safety management.

## 2 Experimental design

### 2.1 Experimental staff and equipment

In an effort to understand the impact of flight simulation on pilot performance, twenty-eight healthy male pilots from the prestigious Civil Aviation Administration of China Flight Academy enthusiastically participated in this comprehensive study. The pilots, with a mean age of 22.5 years (SD = 3.5 years), were right-handed and possessed normal vision and hearing. To ensure the accuracy of the experiment, participants followed strict guidelines during the initial 24-hour period, including abstaining from medications, caffeinated or alcoholic beverages, and ensuring adequate sleep. Additionally, to ensure familiarity with flight simulation skills and equipment operation, participants underwent a rigorous familiarization process with the flight simulation platform prior to the experiment. The experiment commenced at both 10:00 AM and 2:00 PM for all participants. The experiment complied with the Declaration of Helsinki established by the World Medical Association and was approved by the Medical Ethics Committee of Civil Aviation Flight University of China. All participants read and signed the informed consent form before the start of the experiment.

**FIGURE 1:**
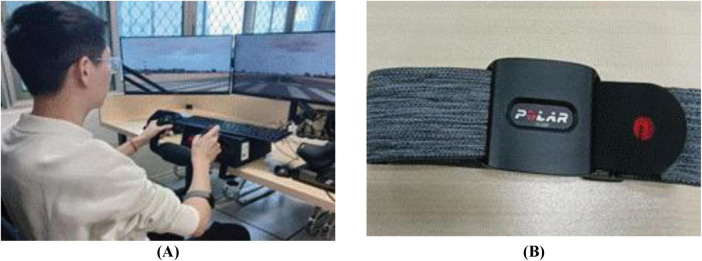
Experimental instruments: **(A)** Flight simulation platform; **(B)** Polar Verity Sense sports armband heart rate monitor.

The experiment used a desktop flight trainer from Microsoft Flight Simulator 2020. This platform, equipped with triple screens, a simulated joystick, a throttle stick, and a braking device, offered a highly realistic flight simulation experience. To continuously monitor the pilot’s HRV in real time, a Polar Verity Sense sports armband HR monitor was chosen for its ease of wear, noninvasiveness, and high accuracy. The simulation equipment and the test environment are depicted in Figure 1.

### 2.2 Experimental task design

The experiment can be divided into two parts: the pre-test and the formal test.

Pre-test: In this phase, subjects are briefed about the experimental objectives, procedures, and mission requirements. They will also familiarize themselves with the flight simulator’s basic operations. During the pre-test, the operator will synchronize the heart rate acquisition equipment to ensure that each subject’s monitor functions correctly. At the end of the pre-test, all subjects will rest for five minutes to stabilize their physiological indicators.

**FIGURE 2:**
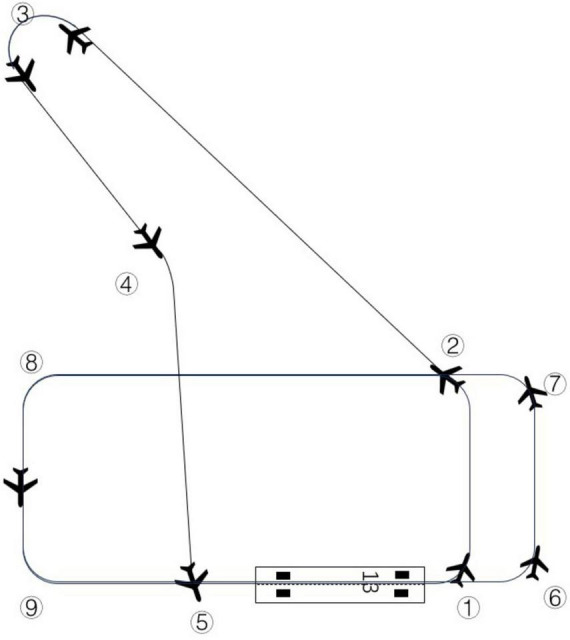
Diagram of the simulated flight path and turn.

Formal Experiment: The simulation is conducted using a Cessna 172 model at the Guanghan Airport, utilizing Runway 13. The weather conditions are simulated with appropriate side winds at a wind speed of approximately 5 knots, and meteorological conditions are CAVOK (clear skies). The flight plan is as outlined below:

1. After takeoff, the heading is 127°, the altitude climbs to 1,800ft, and the flight plan is a 75kt climb.
2. After the first turn, the heading is adjusted to 040°, the altitude is maintained at 3,000ft, and the flight is leveled off at 75 kt.
3. After the second turn, the heading is adjusted to 330°, the altitude is maintained at 4,000 ft, and the speed is adjusted: first down to 65 kt, then up to 95 kt.
4. After the third turn, the heading is adjusted to 150°, the altitude is maintained at 3,000 ft, and the flight is level at 90 kt.
5. After the fourth turn, the heading is adjusted to 180°, the altitude is maintained at 2,500 ft, and the flight is level at 90 kt.
6. After the fifth turn, the heading is adjusted to 127°, the altitude is dropped to 2500 ft, and the flight is level at 90 kt.
7. After the sixth turn, the heading is adjusted to 37°, the altitude is dropped to 2500 ft, and the flight is level at 90 kt.
8. After the seventh turn, the heading is adjusted to 307°, the altitude is dropped to 2400 ft, and the flight descends at 90 kt.
9. After the eighth turn, the heading is adjusted to 217°, altitude is maintained at 2400 ft, and the flight descends at 75 kt.
10. After the ninth turn, the heading is adjusted to 127°, the altitude is dropped to 2200ft, and the flight descends at 75 kt.
11. Finally, the flight entered a stable final approach phase, descending to 65kt, and the runway head speed further descending to 62 kt in preparation for landing. The simulated flight path is shown in Figure 2.

### 2.3 Turning maneuvers with different load

Turn behavior is determined by analyzing changes in heading and altitude data. A 20° or more heading change between consecutive data points is used as the threshold for identifying a turn. The change is identified as a turn when this threshold is met or exceeded. The turn type is further classified based on concurrent altitude data. For example, if the altitude increases during the turn, it is labeled as a climbing turn; if the altitude decreases, it is a decending turn; and if the altitude remains relatively constant, it is identified as a level flight turn.

This study primarily investigates three types of aircraft turning maneuvers: level turns, climbing turns, and descending turns. Each type presents unique challenges and cognitive load for pilots, influenced by the complexity and demands of the maneuvers:

Level Turns: These are the simplest form of the maneuvers mentioned. During a level turn, the pilot only needs to manage the aircraft’s steering (yaw and bank) to change direction while keeping the altitude and throttle settings constant. This requires minimal adjustment beyond steering, making it less cognitively demanding compared to the other types of turns.

Descending Turns: These turns are more complex than level turns. The pilot must adjust not only the steering but also the throttle and altitude. The goal is often to decrease altitude while turning, which involves more control inputs. The throttle may be reduced to manage the descent, and the altitude must be carefully monitored to ensure the aircraft reaches a lower altitude without significant changes in speed.

Climbing Turns: These are the most challenging maneuvers discussed. In a climbing turn, the pilot needs to increase altitude while turning, requiring careful management of the aircraft’s throttle and stick. The throttle must be adjusted to provide enough power for the climb, and the stick must be stabilized to reach and maintain the desired altitude, all while managing the aircraft’s speed to prevent stalling or over-speeding. The trajectory parameters for each turn type are detailed in Table 1. The maneuvers’ complexity is evaluated based on the number of actions required, the intricacy of the actions, and the time constraints involved.

**TABLE 1:** Parameters of the turning phase.

Number	Type	Pre-turn heading/(°)	Post-turn heading/(°)	Heading Delta/(°)	Pre-turn height/(ft)	Post-turn height/(ft)	Speed/(kt)
1	Climbing turn	127	40	87	1800	3000	75
2	Climbing turn	40	330	70	3000	4000	75
3	Leveling turn	330	150	180	4000	4000	90
4	Descending turn	150	180	30	4000	3000	90
5	Leveling turn	180	127	53	2500	2500	90
6	Leveling turn	127	37	90	2500	2500	90
7	Leveling turn	37	307	90	2500	2500	90
8	Descending turn	307	217	90	2500	2400	75
9	Descending turn	217	127	90	2400	2200	75

Through operational complexity and expert scoring, the cognitive load required for these turns ranks as follows: climbing turns > descending turns > level turns.

### 2.4 Experimental procedure

The experiment used a within-subjects, one-way design, where the independent variable was the three turn phases of the flight procedure, and the dependent variable was the heart rate variability (HRV) characteristics. The study included a pre-experiment phase and a formal experiment. During the pre-experiment phase, subjects were equipped with Polar heart rate monitors and familiarized with the flight procedure. In the formal experiment, subjects completed the flight procedure once, with the flight duration varying based on their flight conditions.

## 3 Data processing

### 3.1 Data pre-processing


(1) $$y_{i+k}=y_{i}+\frac{k\,\left(y_{i+n}-y_{i}\right)}{\left(n-1\right)}$$

### 3.2 Characteristic extraction

For the pre-processed PP-interval data, we utilize the HRV analysis library under the Python environment to extract the HRV characteristics. The time window selected is 30 seconds, and the overlap rate of adjacent time windows is 40%. After processing, a total of 30 HRV characteristics, including mean_nni, sdnn, sdsd, pnni_20, rmssd, median_nni, range_nni, cvsd, cvnni, etc., were extracted, as shown in Table 2.

**TABLE 2:** HRV characteristics.

Characteristics	Definition	Characteristics	Definition
mean_nni	Mean value of heartbeat intervals	std_hr	Standard deviation of heart rate
sdnn	Standard deviation of NN intervals	lf	Low frequency power
sdsd	Standard deviation between adjacent NN intervals	hf	High frequency power
pnni_20	NN interval greater than 20 milliseconds per cent	lf_hf_ratio	Ratio of low frequency power to high frequency power
pnni_50	Percentage of NN intervals greater than 20 milliseconds	lfnu	Normalized low frequency power
nni_20	Number of times adjacent NN intervals differ by more than 20 milliseconds	hfnu	Normalized high frequency power
nni_50	Number of times adjacent NN intervals differ by more than 50 milliseconds	total_power	Total energy of the spectrum
rmssd	Root mean square difference between adjacent NN intervals	vlf	Energy of the power spectrum
median_nni	Median of NN intervals	sd1	Standard Deviation 1 in Poincare Plot
range_nni	Range of NN intervals	sd2	Standard deviation 2 in Poincare Plot
cvsd	Coefficient of variation for continuous differences	ratio_sd2_sd1	Ratio of standard deviation 2 to standard deviation 1
cvnni	Coefficient of variation of NN intervals	csi	Sympathetic index
mean_hr	Mean heart rate	cvi	Vagal index
max_hr	Maximum heart rate	Modified_csi	Sympathetic Nerve Index
min_hr	Minimum heart rate	triangular_index	Trigonometric index

### 3.3 Turning load assessment

To enhance the performance and explanatory capabilities of the model, this study utilized numerical analyses and significance screening of key physiological indicators with SPSS 27 software. Initially, the K-S normality test showed that the HRV characteristics of max_hr, lfnu, hfnu, mean_hr, min_hr, pnni_20, cvi, and nni_20 were normally distributed. In contrast, other HRV characteristics demonstrated nonnormal distributions. Subsequent analyses of the distribution of each HRV characteristic under different turning load were performed using both ANOVA and the K-W test, with a significance threshold set at *p* < 0.05. The results, detailed in Tables 3, 4, revealed significant differences in characteristics such as the triangular index, cvsd, rmssd, and sdsd across various flight stages. However, most other HRV characteristics did not exhibit significant differences. Based on these findings, the triangular index, cvsd, rmssd, sdsd, min_hr, pnni_20, and nni_20 were identified as key indicators reflective of the loading degree in different types of turns.

**TABLE 3:** Characteristic K-W test results and average values.

Characteristics	Climbing Turns	Leveling Turns	Descending Turns	Significance
triangular_index	2.572	3.07	2.818	0.005
mean_nni	672.388	702.185	675.061	0.187
ratio_sd2_sd1	1.962	1.82	1.999	0.687
median_nni	669.358	699.177	671.657	0.176
csi	1.962	1.82	1.999	0.687
lf_hf_ratio	2.392	1.802	3.608	0.446
hf	794.54	1643.17	1600.845	0.066
Modified_csi	716.952	678.472	884.728	0.398
vlf	747.216	1241.329	1585.026	0.472
total_power	3412.599	7073.353	9027.519	0.249
std_hr	5.22	6.159	7.07	0.26
lf	1870.842	4188.854	5841.647	0.511
sdnn	48.727	58.045	62.238	0.162
cvsd	0.051	0.068	0.068	0.048
cvnni	0.063	0.079	0.087	0.192
range_nni	190.931	245.873	244.585	0.118
rmssd	37.203	49.198	47.770	0.043
sd2	62.611	73.333	80.048	0.222
sdsd	37.028	49.009	47.556	0.044
nni_20	10.310	12.591	11.096	0.048
sd1	26.646	35.269	34.221	0.043
pnni_50	14.602	19.408	18.330	0.198

**TABLE 4:** Characteristic ANOVA test results and average values.

Characteristics	Climbing Turns	Leveling Turns	Descending Turns	Significance
max_hr	105.617	101.301	106.289	0.095
hfnu	43.849	48.821	46.124	0.368
lfnu	56.151	51.179	53.876	0.368
mean_hr	94.826	89.558	93.049	0.109
min_hr	84.795	75.970	78.903	0.033
pnni_20	35.558	43.429	38.200	0.026
cvi	4.015	4.290	4.256	0.062
nni_20	10.310	12.591	11.096	0.028

Analysis of the HRV characteristics across three types of flight turns—climbing, descending, and leveling—reveals distinctive trends. The indicators such as pnni_20, range_nni, rmssd, sdsd, nni_20, sd1, and triangular_index generally show an upward trend, reflecting increased vagal activity and autonomic nervous system regulation during these phases. Conversely, the min_hr indicator exhibits a downward trend, indicative of the physiological demands and adjustments during the flight. Interestingly, the cvsd indicator did not display a clear trend, possibly due to the limited monitoring time in the turning phase, which may not sufficiently capture significant variations in cvsd. This result corroborates the observed trends where rmssd, pnni_20, nni_20, and min_hr are consistent with past findings, highlighting their reliability as indicators of physiological responses to varying load. HRV serves as a critical marker of the balance between sympathetic and parasympathetic activities, reflecting the individual’s adaptation to load (Hongfang et al., 2016). It is particularly responsive to the psychophysiological changes and fatigue experienced by pilots under dynamic conditions. During high-load conditions, increased release of norepinephrine triggers sympathetic activation, enhancing cardiac contractility and electrical signaling to meet the heightened physiological demands. This sympathetic surge reduces HRV characteristics due to the suppression of the vagus nerve. Conversely, during low-load conditions, parasympathetic activity dominates through acetylcholine release, promoting recovery by decreasing HR, reducing cardiac contractility, and inhibiting cardiac electrical signaling, thus enhancing HRV (Naijing et al., 2022).

## 4 Classification models

Considering the time-series characteristics of HRV during the turn phase of flight trainees, Long Short-Term Memory (LSTM) model effectively identifies and utilizes this time-series information. The addition of the Attention mechanism allows the model to flexibly select and focus on information based on the importance of data at different positions, thereby improving the model’s accuracy. HRV such as triangular_index, cvsd, rmssd, sdsd, min_hr, pnni_20, nni_20, etc., in the turning phase of flight trainees’ flights are identified. A classification model is constructed to accurately categorize the cognitive load of flight trainees during the turn phase in flight simulation.

### 4.1 LSTM algorithm

The LSTM algorithm extracts characteristic information from the time dimension. It sends it to the subsequent network for processing, prediction, and other operations, which is very favorable for the processing of time series (Jiang et al., 2023) at the moment t,


(2) $$f_{t}=\sigma \,\left(W_{f}\cdot \left[h_{t-1},x_{t}\right]+b_{f}\right)$$

(3) $$i_{t}=\sigma \,\left(W_{i}\cdot \left[h_{t-1},x_{t}\right]+b_{i}\right)$$

(4) $$i_{t}=\sigma \,\left(W_{i}\cdot \left[h_{t-1},x_{t}\right]+b_{i}\right)$$

(5) $$C_{t}=f_{t}\,C_{t-1}+i_{t}\,{\tilde{a}}_{t}$$

(6) $$o_{t}=\sigma \left(W_{o}\cdot |h_{t-1},x_{t}|+b_{o}\right)$$

(7) $$h_{t}=o_{t}\,t\,a\,n\,\left(C_{t}\right)$$

Where, *f*_*t*_, *i*_*t*_, *o*_*t*_ is the forgetting gate, input gate, and output gate respectively, *C*_*t*_ is the internal state value of the cell, sigmoid and tanh are the sigmoid activation function The hyperbolic tangent activation function, respectively, *W*_*f*_,*W*_*i*_,*W*_*a*_,*W*_*o*_ is the matrix weights of the forgetting gate, the input gate, the vector of candidate values, and the output gate respectively, and *b*_*f*_,*b*_*i*_,*b*_*a*_,*b*_*o*_ is the corresponding deviation.

### 4.2 Self-Attention mechanism

The Self-Attention mechanism is a variant of the attention mechanism that reduces the reliance on external information, captures the internal relationships between data or characteristics better, and considers all elements in the sequence simultaneously (Zhang et al., 2020). Assume that the hidden layer output vector for one sample of the LSTM network is.


(8) $$H={\left(h_{1},h_{2},h_{3},\cdots ,h_{d}\right)}^{T}$$

Where, *h*_*i*_ ∈ *R^n^*, n is the number of sequential steps of the characteristics.

By randomly initializing the attention mechanism weight matrix w and the bias vector b, which in turn is associated with performing the dot product operation, the importance of the different sequential steps of the ith input characteristic *h*_*i*_ can be expressed as.


(9) $$s_{i}=\phi \,\left(W^{T}\,h_{i}+b\right)$$

Where, *ϕ* (⋅) is the score function.

The normalization operation is performed on it using Softmax to obtain the weight coefficient matrix (Chen et al., 2020).


(10) $$a_{t}=s\,o\,f\,t\,m\,a\,x\,\left(s_{i}\right)=\frac{e\,x\,p\,\left(s_{i}\right)}{\Sigma_{i}\,e\,x\,p\,\left(s_{i}\right)}$$

Put *a*_*t*_ and *H*_*t*_ through the attention mechanism to get the final output vector *V*_*t*_, as shown in the following equation:


(11) $$V_{t}=\Sigma \,a_{t}\,H_{t}$$

The assignment of probability weights is used to improve the model’s evaluation metrics, such as accuracy and F1 value for the unbalanced data in this study.

### 4.3 LSTM-attention model training

**FIGURE 3:**
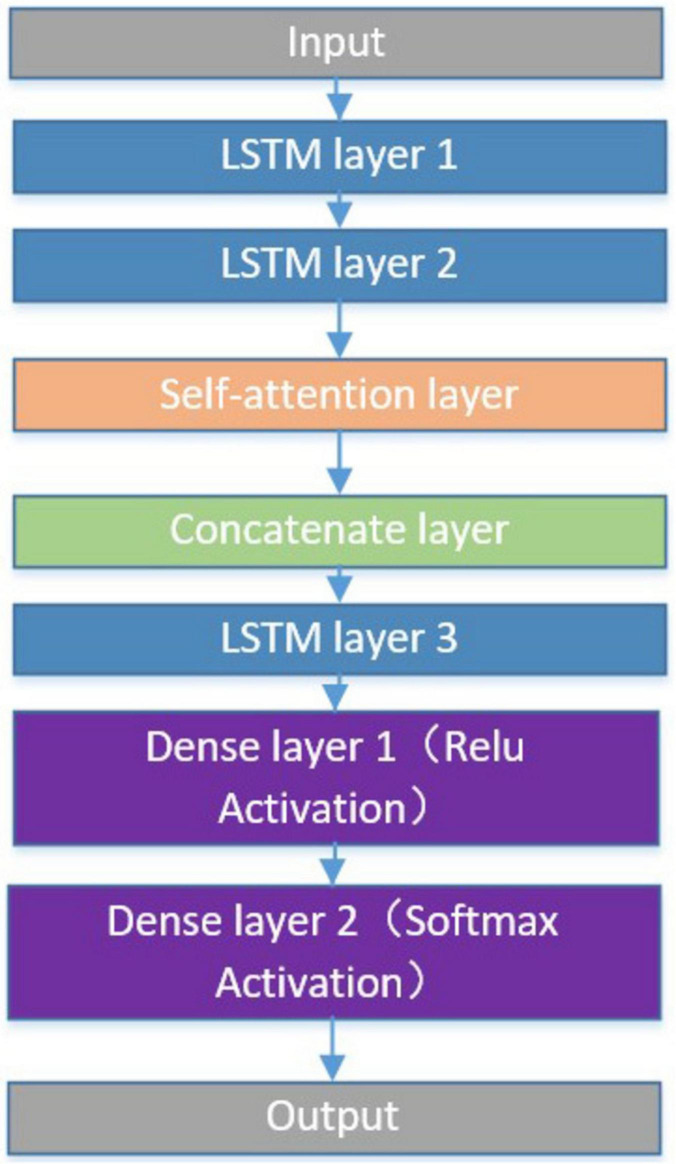
Flowchart of LSTM-Attention model structure.

The LSTM-Attention classification algorithm consists of three LSTM layers, an attention layer, a concatenate layer, and two dense layers. Initially, the model has two LSTM layers, both set to return sequences to allow subsequent layers to process more sequence information. The outputs of these two LSTM layers are fed into a self-attention layer (Attention), which performs feature weighting on the inputs to enhance the model’s ability to capture important information. Next, the output of the Attention layer is merged with the output of the second LSTM layer through a Concatenate layer. After that, the model further processes the merged data through another LSTM layer, which does not return the sequence and only outputs the final result. Finally, the data is processed through two dense layers, the first dense layer using the ReLU activation function and the second dense layer using the softmax activation function for classification, as shown in Figure 3.

During the construction of the LSTM-Attention model, the data were normalized to a range of 0 to 1. The dataset contains labels 1, 2, and 3, corresponding to three different turn types. Up-sampling was used to balance the number of samples in each category to address data imbalance. The data were then divided into training and test sets, with 70% for training and 30% for testing. Each of the first LSTM layers had ten units, while the remaining LSTM layers had 20 units. The fully connected layer had six neurons with the ReLU activation function, and the output layer had three neurons with the softmax activation function.

The Adam optimizer was used during model training with a batch size of 150 and an initial learning rate of 0.005. Training was planned for up to 50 iterations, combined with an early stopping strategy to prevent overfitting. The training would terminate early when performance on the validation set stopped improving. In addition, L2 regularization was added to the LSTM, and the layers were fully connected with a regularization factor set to 0.01.

## 5 Results

**FIGURE 4:**
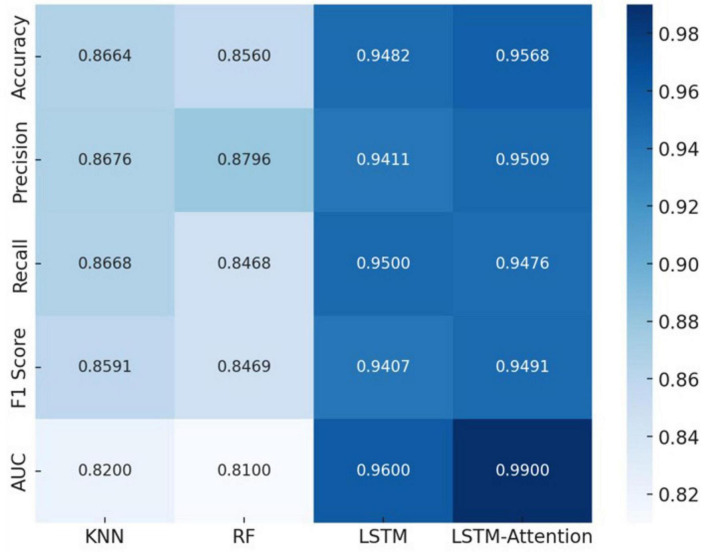
Results of the KNN, RF, LSTM, and LSTM-Attention models.

**FIGURE 5:**
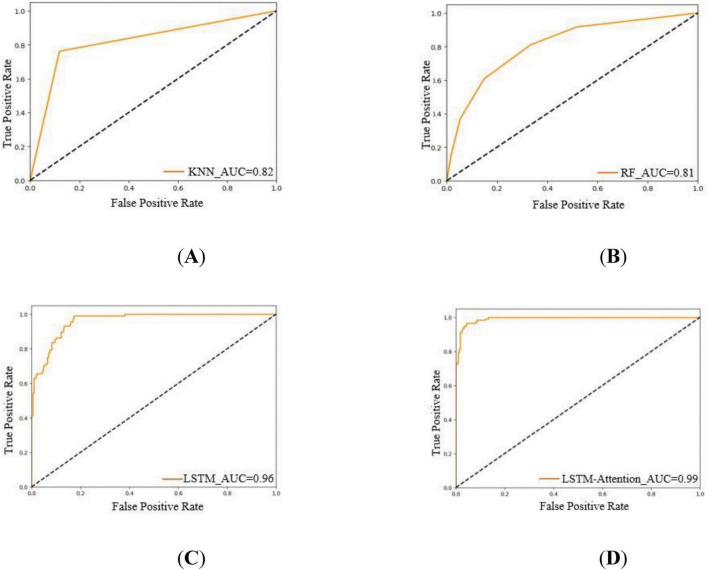
ROC curves and AUC for different models: **(A)** KNN; **(B)** RF; **(C)** LSTM; **(D)** LSTM-Attention.

Various classification algorithms were applied to the extracted HRV dataset to identify flight turns under different loading conditions. The algorithms’ performance was assessed using multiple metrics, including accuracy, precision, recall, and F1 score. The adoption of multiple classification algorithms aimed to thoroughly explore the in-ternal structure and high-dimensional characteristics of the time series data. This approach helps to verify the consistency and reliability of different classification methods in depicting the relationship between HRV and the load of various flight turns. It also aims to elucidate the influence trends of these characteristics while accurately identifying the load status of flight turns. The classification results from multiple machine learning and deep learning classifiers are presented in Figure 4. Additionally, ROC curves and AUC values are utilized to provide a more intuitive evaluation of model performance, as illustrated in Figure 5.

Considering the unevenness of the data and the differences in load levels, the study mainly evaluates the performance of the model in classifying flight turns with different loads based on the F1 scores, and other classification metrics are only for reference (Pereira and Saraiva, 2020). The KNN, RF, LSTM, and LSTM-Attention classifiers show significant differences in the recognition performance among the different turns of the pilots. The LSTM-Attention classifier, with an F1 score of 0.9491, has the best classification performance and the highest accuracy, precision, and recall among all the models, followed by LSTM, which has the highest F1 score. The performance of LSTM closely follows F1 score of 0.9407, while the F1 scores of KNN and RF are 0.8591 and 0.8469, respectively, which are significantly weaker than the previous models. More intuitively, the closer the ROC curve is to the upper left corner, the better the model performance is (Wardhani et al., 2019), The AUC of KNN, RF, LSTM, and LSTM-Attention are 0.82, 0.81, 0.96, and 0.99, respectively, which indicate that LSTM-Attention is the most effective in recognizing the turns of the pilots corresponding to the different load.

## 6 Discussion

Among the classification performances of conventional machine learning models KNN and RF, the KNN model achieves a better F1 score. This suggests that the KNN model is better at capturing the boundary characteristics between different categories in unbalanced data, thus outperforming the RF model in predicting the HRV data in this study. However, due to the small difference between the two in terms of predictive performance metrics, both the KNN and RF methods have significant advantages.

Compared to traditional machine learning methods, the LSTM model demonstrated superior classification results in HRV data for different flight turn phases. This is mainly due to the fact that the LSTM model is able to better handle long-term dependencies and sequence patterns in unbalanced data by using memory cells and gating mechanisms to improve the overall classification accuracy (Coutts et al., 2020). In addition, the LSTM-Attention model with the addition of the self-attention mechanism performs better than the regular LSTM, demonstrating that the self-attention mechanism more accurately captures important patterns and characteristics in the sequences, a result that is consistent with previous findings (Xialin et al., 2022).

Traditional measures of cognitive load rely on human judgment or subjective scales, which often fail to provide the immediate cognitive state of the operator in complex environments. In this study, we assessed the cognitive state of pilots in real-time by exploring the relationship between HRV characteristics and the cognitive load of pilots’ turning behavior. From level flight turning to descend turning to climb turning, the mean values of pnni_20, range_nni, raced, sdsd, nni_20, sd1, and triangular_index characteristic data showed a decrease. The heart rate indicator min_hr showed an increasing trend, suggesting that changes in HRV characteristics and heart rate during changes in cognitive load may be attributed to parasympathetic and sympathetic nerve activity producing corresponding adaptive physiological changes (Naijing et al., 2022; Cao et al., 2019). When performing complex cognitive tasks, the resulting overall decrease in HRV suggests that an individual’s physiological response tends toward a more consistent heart rate pattern, often associated with increased sympathetic activity. This indicates that the body is coping with higher stress or concentration levels. The negative correlation observed shows that as the complexity of cognitive tasks increases, HRV decreases, reflecting the physiological response to heightened cognitive load. For instance, a decrease in pnni_20 may be due to the individual’s need to maintain greater alertness during complex tasks, leading to reduced adaptability in heart rhythm. A decrease in range_nni could result from the body’s response to cognitive challenges, where the heart rhythm stabilizes to ensure effective coordination between the brain and body. SD1 reflects decreased short-term heart rate variability and reduced parasympathetic activity, while a lower Triangular Index indicates a more concentrated heart rhythm with less variability. These trends suggest that as mission difficulty and cognitive load increase, the physiological state of pilots undergoes significant changes to meet higher psychological and physiological demands. Parasympathetic and sympathetic nerves are the two major branches of the autonomic nervous system; parasympathetic nerves contribute to the slowing of the heart rate and the cardiac rhythm to become more stable mainly through the release of acetylcholine, which is manifested as an increase in HRV, and sympathetic nerves respond to emergencies by speeding up the HR through the release of epinephrine and norepinephrine which usually results in a decrease in HRV. During cognitive tasks, especially in response to stress and increased difficulty, sympathetic and parasympathetic nerves interact to produce adaptive changes to regulate physiological and psychological states. It was demonstrated that pilots exhibit significant differences in quantitative indicators of HRV characteristics when performing tasks of varying difficulty (Hajra and Law, 2020). Teachers can use HRV to judge the pilot’s physiological state and adjust the training difficulty to achieve personalized training. Additionally, this finding is similar in other fields, not limited to aviation pilots, such as athletes, civilian police, or hypertensive patients. In these domains, individuals also show significant changes in HRV characteristics when faced with stress (Yijun et al., 2023; Junsen et al., 2022; Munla et al., 2015). By monitoring HRV indicators, instructors can assess pilots’ stress levels and psychological load in real-time, allowing them to adjust the difficulty of training missions accordingly. This real-time monitoring helps prevent incorrect maneuvers caused by excessive pilot stress, enhancing flight safety. Additionally, targeted training enables pilots to manage stress and cognitive load better, improving their performance in complex missions. The present study only used HRV characteristics for pilot cognitive load assessment in a simulated flight environment; however, lacking comparative validation in real environments, its conclusions may differ from reality. Therefore, future studies should include real-flight experiments as well as extensive comparative baselines, such as comparing HRV analyses with other physiological and neuroimaging techniques like electroencephalography and functional magnetic resonance imaging. Furthermore, the sample size should be increased and include pilots with different experience levels and backgrounds (age, sex, etc.) to enhance the generalizability and reliability of the findings (Garavaglia et al., 2021; Schaffarczyk et al., 2022). Such comparisons would not only help to validate the heart rate variability analysis but also further explore the differences in physiological responses to stress and task difficulty among different pilots, enabling more personalized training and support.

## 7 Conclusion

Based on the flight parameters and HRV characteristics obtained from the Simulated Flight experiments, this study classified the load levels corresponding to three types of turns: climbing turns, leveling turns, and descending turns, and analyzed the differences in HRV characteristics under different turn load, which showed that most of the HRV characteristics differed significantly among the three types of turns. The study shows that most HRV characteristics are significantly different among the three types of turns. The load level is relatively high for climbing turns, lower for descending turns, and lowest for leveling turns.

The LSTM-Attention model performs the best in identifying the cognitive load levels of different turns, surpassing the traditional machine learning model and the model without attention mechanism, and can effectively identify and classify the cognitive load levels of different turns, which is conducive to the optimization of load allocation. It can help flight schools, airlines, and general aviation companies to make reasonable load assignments inflight tasks and schedules with different turn demands.

## Data Availability

The datasets presented in this article are not readily available because the datasets presented in this article were aimed at pilots. For privacy reasons, they are not readily available. Requests to access the datasets should be directed to a3224589424@163.com.
